# First report of cestode infection in the crustacean *Artemia persimilis* from Southern Chilean Patagonia and its relation with the Neotropical aquatic birds

**DOI:** 10.7717/peerj.7395

**Published:** 2019-08-06

**Authors:** Stella Redón, Gergana P. Vasileva, Boyko B. Georgiev, Gonzalo Gajardo

**Affiliations:** 1 Laboratory of Genetics, Aquaculture & Biodiversity, Universidad de Los Lagos, Osorno, Región de Los Lagos, Chile; 2 Institute of Biodiversity and Ecosystem Research, Bulgarian Academy of Sciences, Sofia, Bulgaria

**Keywords:** Brine shrimp, South American flamingos, Hypersaline wetlands, Cestodes, Chilean patagonia, Biodiversity, Hymenolepididae

## Abstract

The brine shrimp genus *Artemia* Leach (Crustacea, Branchiopoda), a keystone group in hipersaline wetlands all over the world, offers an excellent model to study species interactions (parasitism) and to explore “hidden fauna” (avian endoparasites). The present study is the first report on the parasite infection of the South American species* Artemia persimilis* from the Southern Chilean Patagonia (50°S–53°S). Samples were collected in Los Cisnes and Amarga lagoons, the two most austral populations of this crustacean described to date, during two seasons (spring and autumn). A total of 98 larvae of cestodes of the family Hymenolepididae (Cestoda, Cyclophyllidea) were found and identified as belonging to the following taxa: *Confluaria podicipina* (adult parasitic in grebes), *Flamingolepis* sp. (a cestode parasite of flamingos), *Fimbriarioides* (?) sp. (adults of the species of this genus infect waterfowl and shorebirds) and *Wardium* sp. (definitive host unknown, most probably charadriiform birds). This is a new geographical record of *C. podicipina* and the genus *Fimbriarioides* for the Neotropical Region, the latter being the most widely distributed species at both localities and seasons surveyed, and the only species recorded in autumn (April). Cestode community composition in Los Cisnes population was characterised by dominance of *Flamingolepis* sp., representing more than 65% of the total cestode species recorded, whereas in the Amarga population the most abundant parasite (>83%) was *Fimbriarioides* (?) sp. Significant seasonal variations were detected in Los Cisnes lagoon for *Flamingolepis* sp. and *C. podicipina*, with exclusive presence of them in spring (November). Besides providing novel information on cestodes infection in *A. persimilis,* this study provides new data on the life cycle of cestodes of Neotropical aquatic birds such as South American flamingos and grebes. Our finding expands the knowledge on the biodiversity and population dynamics of extreme and unique environments from high latitudes (Patagonia) and makes evident the need of further taxonomical and ecological studies for better understanding the life cycles of avian helminth parasites in the Neotropics and the role of aquatic invertebrates in them.

## Introduction

Conservation of biodiversity is one of the greatest challenges in this century given the accelerated rate of species loss due to global threats such as habitat degradation, biological invasions, overexploitation and co-extinctions. Parasites are important components of biodiversity and they are exposed to the same threats as their hosts (Gómez & Nichols, 2013). However, maintenance of the parasite biodiversity has not been traditionally considered as a priority strategy (Dunn et al., 2009) despite the fundamental role of parasitic organisms as ecological and evolutionary drivers, biomarkers of the ecology of their hosts and of ecosystems health as well as the benefits they can provide for host populations (reviewed by Gómez & Nichols, 2013). Understanding the host-parasite relationships in a given ecosystem, either in terms of the host specificity, virulence, transmission pathways or life cycles, is essential to know and preserve its biodiversity. Parasites with complex life cycles, i.e., those that involve more than one host, which are usually part of a common food web, may be used as good indicators of the aquatic biodiversity (Hechinger & Lafferty, 2005) and can reflect the specific diet of the final host and their population dynamics.

Hypersaline wetlands (i.e., salinity > 50 g/L; *sensu* Hammer, 1986) are natural laboratories for biodiversity key-studies due to their relatively simple trophic webs (Gajardo, Sorgeloos & Beardmore, 2006). The brine shrimps of the genus *Artemia* Leach (Crustacea: Branchiopoda: Anostraca) have a main ecological role in hypersaline ecosystems, both as food resource for aquatic bird communities (Sánchez, Green & Castellanos, 2006; Varo et al., 2011) and as filter feeders controlling the primary production via regulating the abundance of phytoplankton and transparency of water column, with potential for cascading effects (Mohebbi, 2010; Belovsky et al., 2011; Sánchez et al., 2016). Furthermore, they act as intermediate hosts of helminth parasites of aquatic birds such as flamingos, grebes, gulls, shorebirds and ducks (Georgiev et al., 2005; Vasileva et al., 2009; Redón et al., 2015a). Previous studies on helminths of *Artemia* spp. from the Western Mediterranean and USA have demonstrated the participation of brine shrimps in the life cycles of 15 cestode species of the order Cyclophyllidea and unidentified nematode species of the family Acuariidae (Georgiev et al., 2005; Vasileva et al., 2009; Redón et al., 2015a).

Two species of *Artemia* have been described as native for the American continent: *A. franciscana* Kellogg, widely distributed across the continent, and *A. persimilis* Piccinelli & Prosdocimi, restricted to Argentina and Southern Chile (Triantaphyllidis, Abatzopoulos & Sorgeloos, 1998; De los Ríos-Escalante, 2013). Information about their parasites is rather limited; the only comprehensive study has demonstrated the participation of *A. franciscana* in the circulation of helminth parasites in the Great Salt Lake (Utah, USA) (Redón et al., 2015a). For South American *Artemia* populations, their role in the transmission of avian parasites has never been explored. The aim of the present study is to fill the gap of knowledge by presenting the first helminthological study of *A. persimilis* from Southern Chilean Patagonia, including spatiotemporal effects on the dynamics of infection. The sampling sites include two of the most austral populations of *A. persimilis* described to date in Chile (Gajardo et al., 2002; De los Ríos-Escalante, 2013).

## Material and Methods

### Study area and *Artemia* sampling

**Figure 1:**
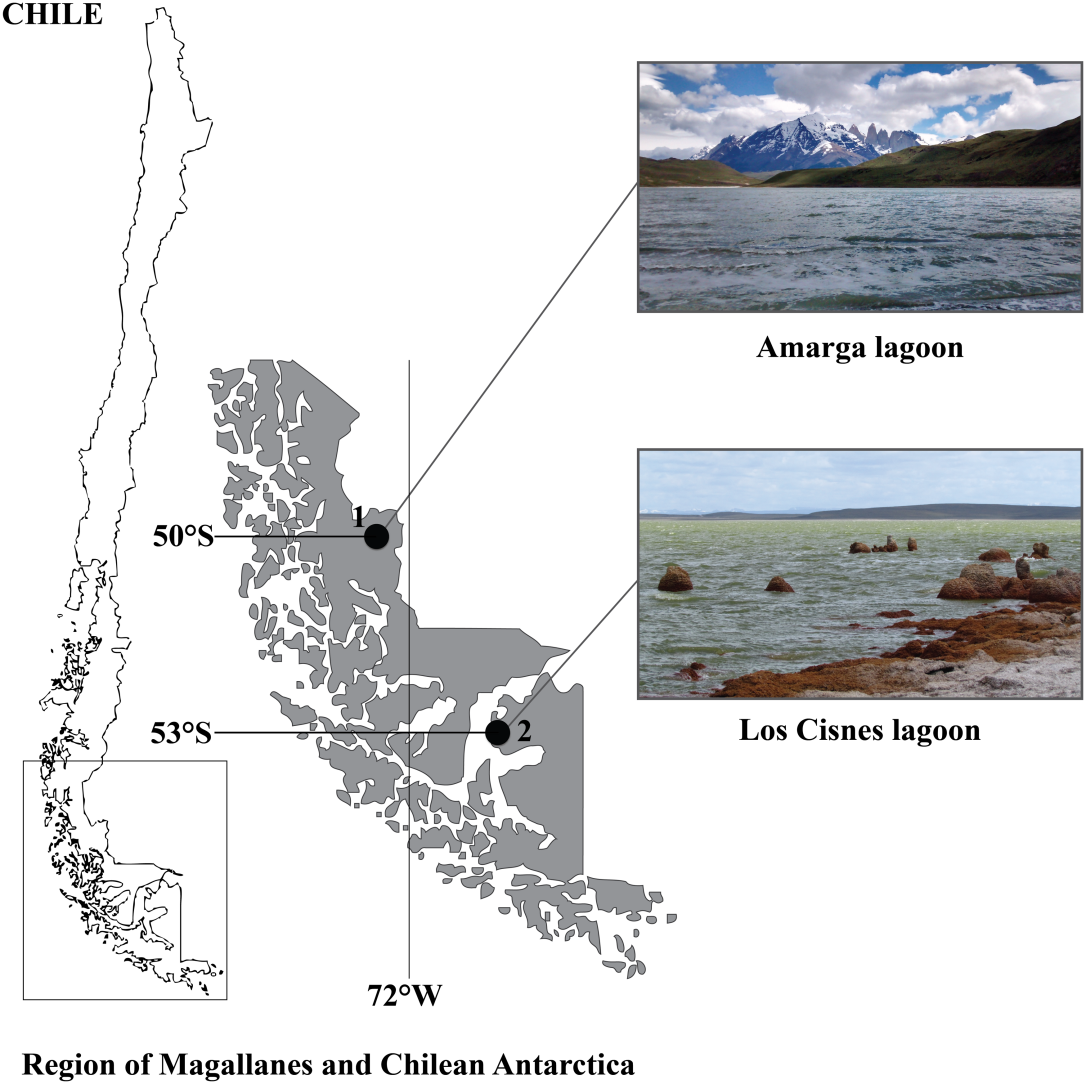
Geographical distribution of the study sites in the Region of Magallanes and Chilean Antarctica. (1) Amarga lagoon. (2) Los Cisnes lagoon.

The study area comprises two hypersaline aquatic ecosystems in the Southern Chilean Patagonia (50°–53°S), i.e., the Region of Magallanes and Chilean Antarctica (Fig. 1). Los Cisnes lagoon (53°15′S, 70°22′W) is located on Tierra del Fuego Island close to its main town Porvenir. It occupies 25.3 ha and represents an important habitat for flamingos, swans, grebes and shorebirds. In 1982, it was declared Natural Monument to protect its high diversity of aquatic birds (c. 50 species, see CONAF, 2009; CONAF, 2014). Amarga lagoon (50°58′S, 72°43′W) is a lake of 2.5 km length, 1.1 km breadth and 2.6 m of depth (Campos et al., 1996). It is located in the Province of Última Esperanza, at the entrance of Torres del Paine National Park. This Biosphere Reserve harbours a great diversity and abundance of birds, with c. 118 avian species using the area as permanent residents or migratory species, including flamingos, ducks, grebes, swans and geese (Matus & Barría, 1999; CONAF, 2007).

The local populations of *Artemia* were identified as *A. persimilis*, a species previously considered endemic to Argentina (Triantaphyllidis, Abatzopoulos & Sorgeloos, 1998) but later discovered in the Southern Chile, i.e., in the Amarga lagoon (Gajardo et al., 1998) and in Los Cisnes lagoon (De los Ríos-Escalante, 2010). Our sampling was carried out in spring (November 2017) and autumn (April 2018). Salinity of the water was measured *in situ* with a portable refractometer ATAGO^®^. *Artemia* individuals were collected with a plankton hand net (180 µm mesh size), washed in fresh water to remove salt and fixed in 70% ethanol. *Artemia* samples were transported to the laboratory and preserved at 4 °C for future parasitological examination. Additional living specimens were transferred into plastic bottles of 5 l containing lake brine and transported to the laboratory for further examination. Field work was conducted under an authorization for research activities in protected wild areas (Permission No 025/2017) issued by the Department of Conservation of Biological Diversity, National Forest Corporation of Chile (CONAF).

### Processing brine shrimp samples and helminth identification

A total of 800 brine shrimps (200 individuals per site and season, mostly adults), randomly selected, with sex ratio c. 1:1, were mounted in glycerol and examined under a compound light microscope Olympus BX50 for the presence of cestodes. After screening, some cysticercoids (i.e., the larval stage of cestodes developing in the intermediate host) were isolated and mounted as microscope slides in Berlese’s medium for a more detailed morphological examination and taxonomic identification. In addition to these quantitative samples, in order to base the morphological examinations and the identification on greater numbers of parasite specimens, we examined some further *Artemia* individuals; the number of the cysticercoids recorded is given below in the text for each species. However, these additional cysticercoids were not used for the calculation of the infection parameters, which were based on the quantitative samples only.

Identification of cysticercoids was based on a comparison with the previous descriptions of cestode larvae that use branchiopods as intermediate hosts (Maksimova, 1973; Maksimova, 1976; Maksimova, 1981; Maksimova, 1989; Gvozdev & Maksimova, 1978; Georgiev et al., 2005; Vasileva et al., 2009; Redón et al., 2015a). Terminology of the cysticercoids follows Chervy (2002). The morphological examination of parasites (including measuring and preparation of drawings and photographs) was carried out using the facilities of IBER-BAS, Sofia, Bulgaria. Metrical data are given as a range, followed by the mean and number of observations (n) in parentheses. The measurements are in micrometres unless otherwise stated. Photographs were taken using Zeiss Axio Imager 2 light microscope equipped with differential interference contrast (DIC) and Jenoptik ProgRes® microscope camera incorporated. Drawings were prepared using Olympus BX51 microscope equipped with a drawing tube. The opensource graphic software, GIMP and Inkscape, were used for the image processing of illustrations.

Voucher specimens of cysticercoids have been deposited at the Invertebrate Collection of the Natural History Museum in Geneva (MHNG), Switzerland.

The specimens studied for each species were as follows:

– *Confluaria podicipina:* Los Cisnes lagoon, 29 November 2017, 5 cysticercoids, isolated and mounted in Berlese’s medium; MHNG-PLAT-122057, a cysticercoid isolated and mounted in Berlese’s medium; Amarga lagoon, 26 November 2017, 2 cysticercoids, one of them isolated and mounted in Berlese’s medium.
– *Fimbriarioides* (?) sp.: Los Cisnes lagoon, 29 November 2017, 6 cysticercoids, 17 April 2018, 7 cysticercoids; all cysticercoids mounted and measured in glycerol, subsequently 7 cysticercoids isolated and mounted in Berlese’s medium; MHNG-PLAT-122058, a cysticercoid isolated and mounted in Berlese’s medium; Amarga lagoon, 26 November 2017, 3 cysticercoids, 27 November 2017, 2 cysticercoids, 18 April 2018, 1 cysticercoid; all cysticercoids mounted and measured in glycerol, subsequently 2 cysticercoids isolated and mounted in Berlese’s medium.
– *Flamingolepis* sp.: Los Cisnes lagoon, 29 November 2017, 14 cysticercoids, mounted and measured in glycerol; subsequently 12 cysticercoids isolated and mounted in Berlese’s medium; MHNG-PLAT-122059, a cysticercoid isolated and mounted in Berlese’s medium.
– *Wardium* sp.: Los Cisnes lagoon, 29 November 2017, 2 cysticercoids mounted and measured in glycerol; subsequently both cysticercoids isolated and mounted in Berlese’s medium.

### Parameters of infection and statistical analysis

Infection descriptors for each cestode species and for the overall infection were calculated according to Bush et al. (1997): prevalence (P%, number of infected individuals of the host population presented as percentage of the total number of examined individuals), mean intensity (MI, mean number of parasites per infected individual of the host population) and mean abundance (MA, mean number of parasites per examined individual of the host population, both infected and non-infected) In order to characterise the cestode community composition for each locality and season, we calculated the relative abundance (RA%, percentage of the number of individuals of a certain parasite species from the total number of individuals of all parasite species in the examined host population) for each parasite species.

Generalized linear models (GLMs) were performed to analyse prevalence of infection (total cestodes and *Fimbriariodes* (?) sp.) in relation to locality (Los Cisnes and Amarga lagoons), season (spring and autumn) and interactions between both factors. For *Confluaria podicipina*, we tested only the effect of the locality since this species was exclusively recorded in spring. A binomial error distribution and log link function were used in the model. GLM analyses were not performed for *Flamingolepis* sp. and *Wardium* sp. due to a quasicomplete separation of the observed data since infection was only detected in one locality and in one season. Instead, *Z* tests were used to analyse differences in the prevalence of *Flamingolepis* sp. between seasons and between localities. However, no statistical analyses were applied for *Wardium* sp. because only one infected individual was found. Non-parametrical Mann–Whitney *U* test was applied for testing temporal variations on the abundance of parasites and the intensity of infection. The same procedure was applied to analyse variations of these infection parameters between the localities within the same season. Non-parametric statistics were used owing to the lack of normality in the distributions of these parameters (even after transformations). Significance was assumed at *p* ≤ 0.05. Statistical analyses were performed using SPSS 15.0 for Windows (SPSS Inc. Chicago, IL, USA).

## Results

### Identification of helminth larvae

In the studied brine shrimps, we found larvae (cysticercoids) of cestodes belonging to four species and four genera of the family Hymenolepididae. They were identified at either the species level or the generic level on the basis of their morphology. The morphological characters of these larvae as well as the argumentation of our taxonomic identifications and possible associations with avian definitive hosts are presented in the following systematic survey.

**Figure 2:**
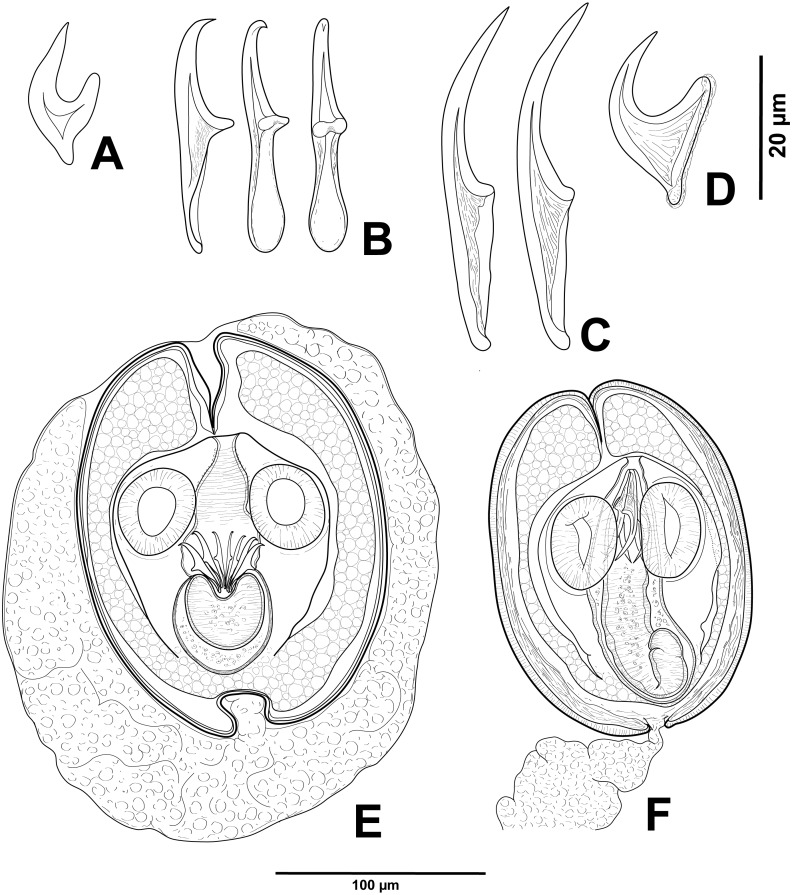
Cestode larvae from *Artemia persimilis*, Amarga lagoon (A, E) and Los Cisnes lagoon (B–F), Chile. (A) Hook of *Confluaria podicipina* (Szymanski, 1905). (B, E) Hooks and cysticercoid of *Fimbriarioides* (?) sp. (C, F) Hooks and cysticercoid of *Flamingolepis* sp. 1. (D) Hook of *Wardium* sp. Drawing credit: Gergana P. Vasileva.

**Figure 3:**
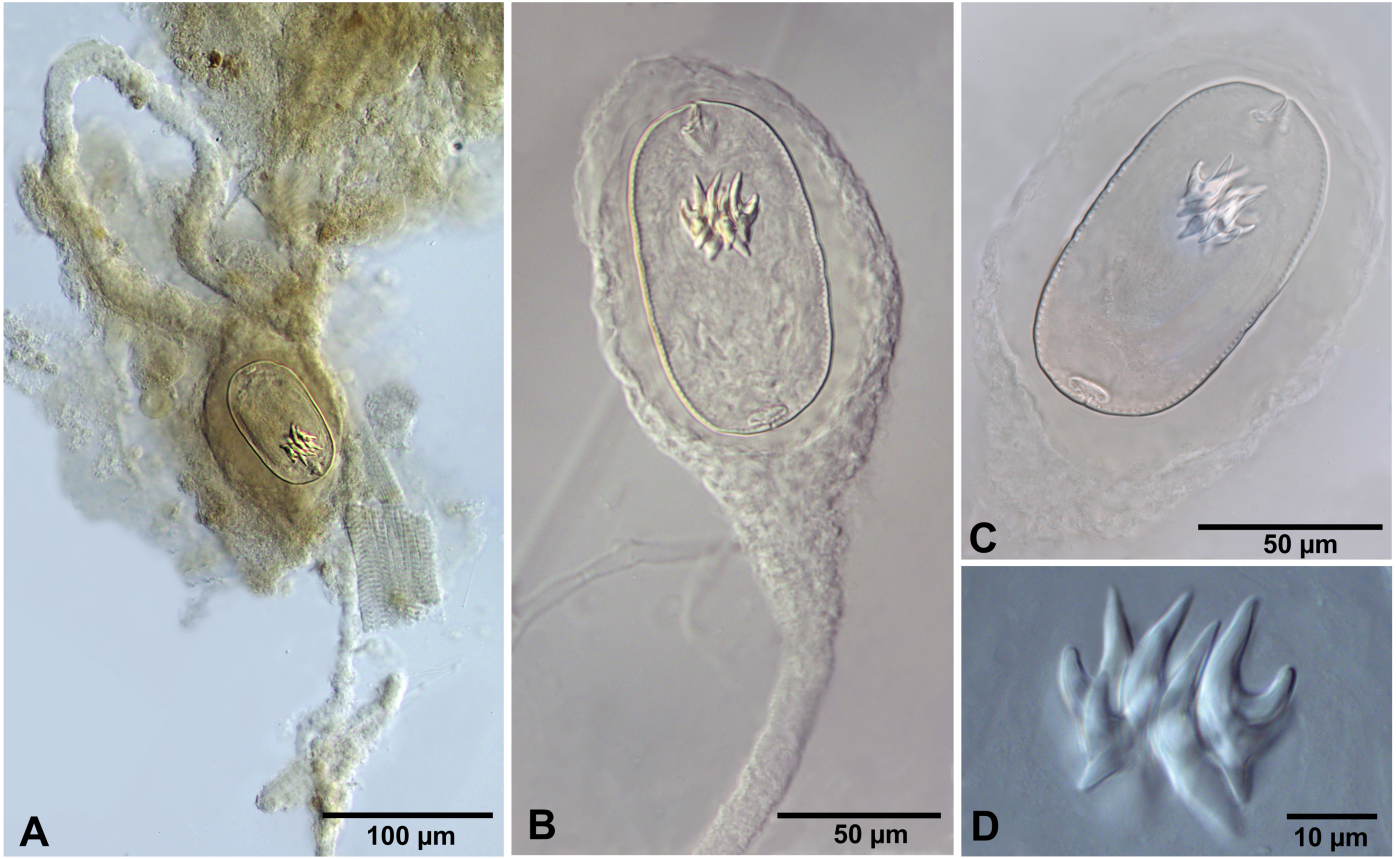
*Confluaria podicipina* (Szymanski, 1905) from *Artemia persimilis*, Los Cisnes lagoon (A) and Amarga lagoon (B–D), Chile (mounts in Berlese’s medium). (A) Isolated cysticercoid with unpacked cercomer. (B) Fully developed cysticercoid with a part of unpacked cercomer. (C) Internal cysts. (D) Rostellar hooks.

***Confluaria podicipina* (Szymanski, 1905) (Figs. 2A, 3)****

#### Remarks

We do not provide a complete description of cysticercoids from *A. persimilis* from Chile because they have morphological characters similar to those previously described for *C. podicipina* from Central Asia, Europe and North America (see Maksimova, 1981; Georgiev et al., 2005; Redón et al., 2015a): presence of an external envelope formed by the anterior part of the cercomer (Figs. 3A, 3B); invaginable rostellum armed with 10 aploparaksoid hooks (Figs. 2A, 3C, 3D); a very long and coiled cercomer, densely packed in a thin membranous envelope (Fig. 3A). The measurements of isolated cysticercoids in Berlese’s medium corresponded well to the previous data from *A. parthenogenetica* in Spain (Georgiev et al., 2005) and *A. franciscana* in USA (Redón et al., 2015a) (see Table 1). The only exception was the size of the external capsules of the cysticercoids, which was smaller in the specimens from Chile. Our results revealed less variations in the total size of the external capsule in the samples of *C. podicipina* from *A. persimilis* in comparison to those from USA and Spain, where we found cysticercoids of various sizes, including some specimens with intermediate measurements (Redón et al., 2015a). This difference was probably due to the smaller sample size of this parasite species in the present study. Despite the differences in the size of the external capsule, the morphology of the cysticercoids, the shape of their rostellar hooks and the remaining metrical data were similar to those reported in previous descriptions. On this basis, we identified these cysticercoids from *A. persimilis* as *C. podicipina*.

**Table 1:** Metrical data of cysticercoids of *Confluaria podicipina* (Szymanski, 1905) recorded in *Artemia* spp. from various localities. All measurements in µm.

Host		*A. parthenogenetica*			*A. franciscana*			*A. persimilis*		
Locality		Odiel marshes (Spain)			Great Salt Lake (USA)			Los Cisnes lagoon and Amarga lagoon (Chile)		
References		Georgiev et al. (2005)			Redón et al. (2015a)			Present study		
		Range	Mean	*n*	Range	Mean	*n*	Range	Mean	*n*
External	length	195–255	215	9	144–313	212	15	113–185	135	5
capsule	width	135–204	152	9	72–192	118	15	73–110	87	5
Cyst	length	93–147	121	12	72–143	106	17	80–100	90	7
	width	47–87	73	12	36–89	61	17	43–55	49	7
Scolex	length	72–104	92	12	38–52	48	15	50–63	57	6
	width	38–72	59	12	25–47	37	15	33–40	38	6
Suckers	diameter	26–32	28	10	21–26	22	6	16–23	19	10
Rostellum	length	42–47	46	7	23–31	27	10	25	25	3
	width				–	–	–	18–23	20	3
Rostellar	total length	21–24	22	12	19–21	20	11	18–21	19	10
hooks	blade	–	–	–	11	–	11	10–12	11	10
	handle	–	–	–	3–5	4	11	3–4	4	10
	guard	–	–	–	5–6	6	11	5–6	6	10

The species of the genus *Confluaria* Ablasov in Spasskaya, 1966 are specific parasites of grebes (Podicipediformes), recorded mostly from the Holarctic Region (Vasileva, Georgiev & Genov, 1999a; Vasileva, Georgiev & Genov, 1999b; Vasileva, Georgiev & Genov, 2000; Vasileva, Kornyushin & Genov, 2001; Vasileva, Skirnisson & Georgiev, 2008; Sitko & Heneberg, 2015). In South America, there are only two records of *Confluaria* spp.: *C. capillaris* (Rudolphi, 1810) in *Tachybaptus dominicus* (L.) in Brazil (Fuhrmann, 1906; Vasileva, Georgiev & Genov, 1999a) and an identified species of *Confluaria* reported from the Southern Silvery Grebe *Podiceps occipitalis* Garnot in the central Chile (González-Acuña et al., 2017). Until now, *C. podicipina* has been recorded in the Holarctic Region (Stock & Holmes, 1987; Vasileva, Georgiev & Genov, 2000). In North America (Alberta, Canada), *Podiceps auritus* (L.) and *P. nigricollis* have been reported as definitive hosts of *C. podicipina* (Stock & Holmes, 1987) and its confirmed intermediate host is *A. franciscana* (Redón et al., 2015a).

The present study is the first report of this species in the Neotropical Region and might be associated with grebe species distributed in Southern Patagonia. These are Silvery Grebe *Podiceps occipitalis*, Hooded Grebe *Podiceps gallardoi* Rumboll, White-tufted Grebe *Rollandia rolland* (Quoy & Gaimard), Pied-billed Grebe *Podylimbus podiceps* (L.) and the Great Grebe *Podiceps major* (Boddaert) (see Sylvester, 1995; Storer, 2000; CONAF, 2014). Based on its anatomical peculiarities (Fjeldså, 1983; Storer, 2000), feeding behaviour (Fjeldså, 1981) and diet (Wetmore, 1926), the most probable definitive host of *C. podicipina* in Patagonian lakes is *P. occipitalis*. The biological traits of this grebe species allow brine shrimps to be essential part of its feeding resources and it is the South-American grebe with dietary peculiarities similar to those of *P. nigricollis* (Mahoney & Jehl Jr, 1985; Storer, 2000). In contrast, *Podylimbus podiceps* feeds on crabs, crayfish and other hard-bodied animals; *Rollandia rolland* is a generalist, feeding on oligochaetes, crustaceans, fish and molluscs; *Podiceps major* is fish-eating; and *P. gallardoi* feeds on rather big amphipods, leaches and especially snails (Fjeldså, 1983; Storer, 2000).

**Figure 4:**
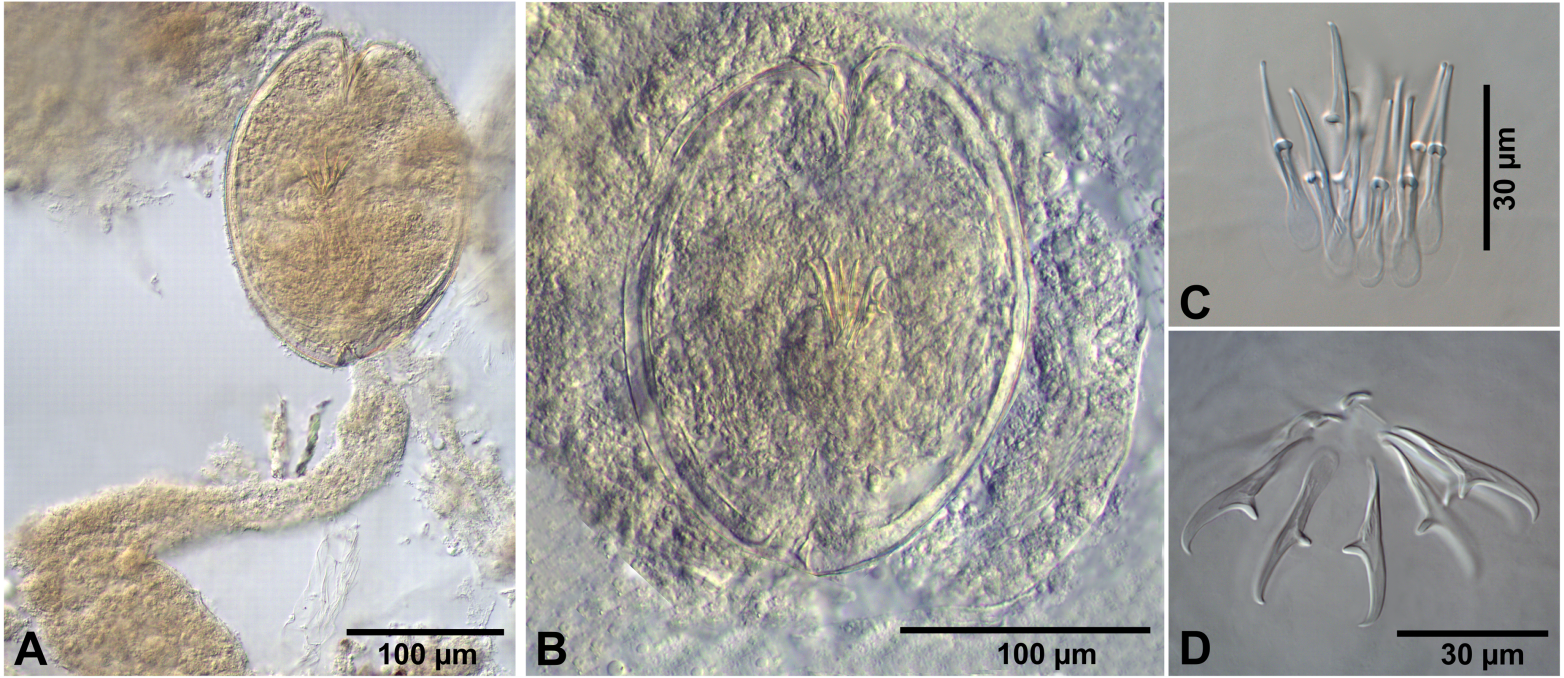
*Fimbriarioides* (?) sp. from *Artemia persimilis*, Los Cisnes lagoon (A, D) and Amarga lagoon (B, C), Chile. (A) Cysticercoid with smashed outer cyst and unpacked cercomer (temporary glycerol mount). (B) Acaudate diplocyst with fully developed cysticercoid (temporary glycerol mount). (C, D) Rostellar hooks of isolated cysticercoids (mounts in Berlese’s medium).

***Fimbriarioides* (?) sp. (Figs. 2B, 2E, 4)****

*Description of cysticercoids* **** (metrical data based on specimens mounted in Berlese’s medium): Diplocysticercoid. Outer envelope thin-walled, formed by packed cercomer, usually encircling internal cyst, often with irregular shape (Figs. 2E, 4B). Internal cyst lemon-shaped, thick-walled. Scolex almost oval, 195–210 ×163 − 195 (203 × 170, *n* = 5), with short apical protrusion. Suckers oval, muscular, unarmed; diameter 45–65 (56, *n* = 11). Rhynchus thick-walled, with distinct radial musculature (Fig. 2E). Rostellar sheath sac-like, 95–100 ×53 (98 ×53, *n* = 2); thin-walled (Fig. 2E). Rostellum invaginable, cup shaped, muscular, with apical invagination. Rostellar hooks resembling diorchoid, 10 in number. Each hook with long and straight handle with flattened, spatulate posterior end (Figs. 2B, 4C, 4D); blade sickle-shaped, slightly shorter than handle; guard short, with slightly bifurcated thickening (Figs. 2B, 4C). Measurements of rostellar hooks: total length 32–35 (33, *n* = 10), length of blade 13–15 (14, *n* = 10), length of handle 16–20 (17, *n* = 10), length of guard 3–4 (4, *n* = 10). Calcareous corpuscles concentrated anteriorly to scolex. Cercomer thick, long, usually forming compact additional thin-walled envelope surrounding cyst; unpacked cercomer thin-walled (Fig. 4A).

*Additional measurements based on specimens in temporary glycerol mounts*: diplocyst with outer envelope 200–315 ×155–265 (254 ×209, *n* = 6); internal cyst 175–215 ×130 − 155 (192 ×151, *n* = 13); scolex 118–130 ×98 − 125 (124 ×112, *n* = 9); diameter of suckers 38–53 (46, *n* = 21); rostellar sheath 75–108 ×45 − 60 (99 ×52, *n* = 7), rostellum 45–58 ×33 − 48 (49 ×41, *n* = 8); longest fragment of cercomer 470 ×88.**

#### Remarks

There are 15 valid genera of the family Hymenolepididae characterised with 10 diorchoid rostellar hooks and parasitic in birds (see Czaplinski in Czaplinski & Vaucher, 1994; Mariaux et al., 2017). Among them, three genera possess invaginable rostellum, i.e., *Fimbriaria* Fröhlich, 1802, *Fimbriarioides* Fuhrmann, 1932 and *Fimbriasacculus* Alexander & McLaughlin, 1996 (see Czaplinski & Vaucher, 1994; Alexander & McLaughlin, 1996). They belong to the subfamily Fimbriariinae Wolffhügel, 1898 and include parasites of aquatic birds (mainly Anseriformes and Charadriiformes), accidentally Galliformes (Spasskaya, 1966; Czaplinski in Czaplinski & Vaucher, 1994).

The hooks of the cysticercoids from *A. persimilis* in Chile are characterised by a sickle-shaped blade, slightly shorter than the handle, a spatulate posterior end of handle and a slightly bifurcated thickening of the guard. This shape differs from the hooks of *Fimbriaria* spp., which have blades much shorter than handle and their guards are not bifid; a spatulate handle has been illustrated only for *F. czaplinskii* Grytner-Zięcina, 1994 (see Fig. 4 of Cielecka et al., 2011). In addition, data about the life cycle of *Fimbriaria* spp. indicate that they have freshwater life cycles that include mostly copepods, ostracods and amphipods as intermediate host (Jarecka, 1958; Spasskaya, 1966).

The hooks of the cysticercoids from *A. persimilis* resemble those of the species of *Fimbriarioides* (see Webster, 1943; Maksimova, 1976; Maksimova, 1989) and *Fimbriasacculus* (see Alexander & McLaughlin, 1996). Webster (1943) reported the presence of “long, bifid guard” in *Fimbriarioides haematopodis* Webster, 1943 and *F. lintoni* Webster, 1943. Maksimova (1989) also mentioned a peculiar structure of the rostellar hooks of *F. spasskyi* Maksimova, 1989, in which the blade and the guard form a “clamp-like” anterior part of the hook. A spatulate posterior end of the handle has been illustrated for two species of *Fimbriarioides*, i.e., *F. tadornae* Maksimova, 1976 (see Vasileva et al., 2009) and *F. spasskyi* as well as for *Fimbriasacculus africanensis* Alexander & McLaughlin, 1996 described from three species of Anatinae in South Africa (see Fig. 5 of Alexander & McLaughlin, 1996). Unfortunately, the generic differentiation between *Fimbriarioides* and *Fimbriasacculus* is based mainly on the strobilar morphology, which is not applicable to our study. There are no published records of species belonging to these genera from waterfowl in South America. By this reason, the generic identification of our larvae is somewhat conditional. We prefer to keep the identification of cysticercoids from *A. persimilis* as “*Fimbriarioides* (?) sp.” by two reasons: (i) the hooks of *Fimbriasacculus* have no bifurcation of the guard; (ii) the previous data on the life cycles of *Fimbriarioides* spp. confirm the role of branchiopods as their intermediate hosts (Maksimova, 1976; Vasileva et al., 2009). Another reason could be the fact that similar cysticercoids, i.e., diplocysticercoid (Chervy, 2002) have been recorded for *Fimbriarioides intermedia* (Fuhrmann, 1913) (see Belopolskaya, 1953).

**Figure 5:**
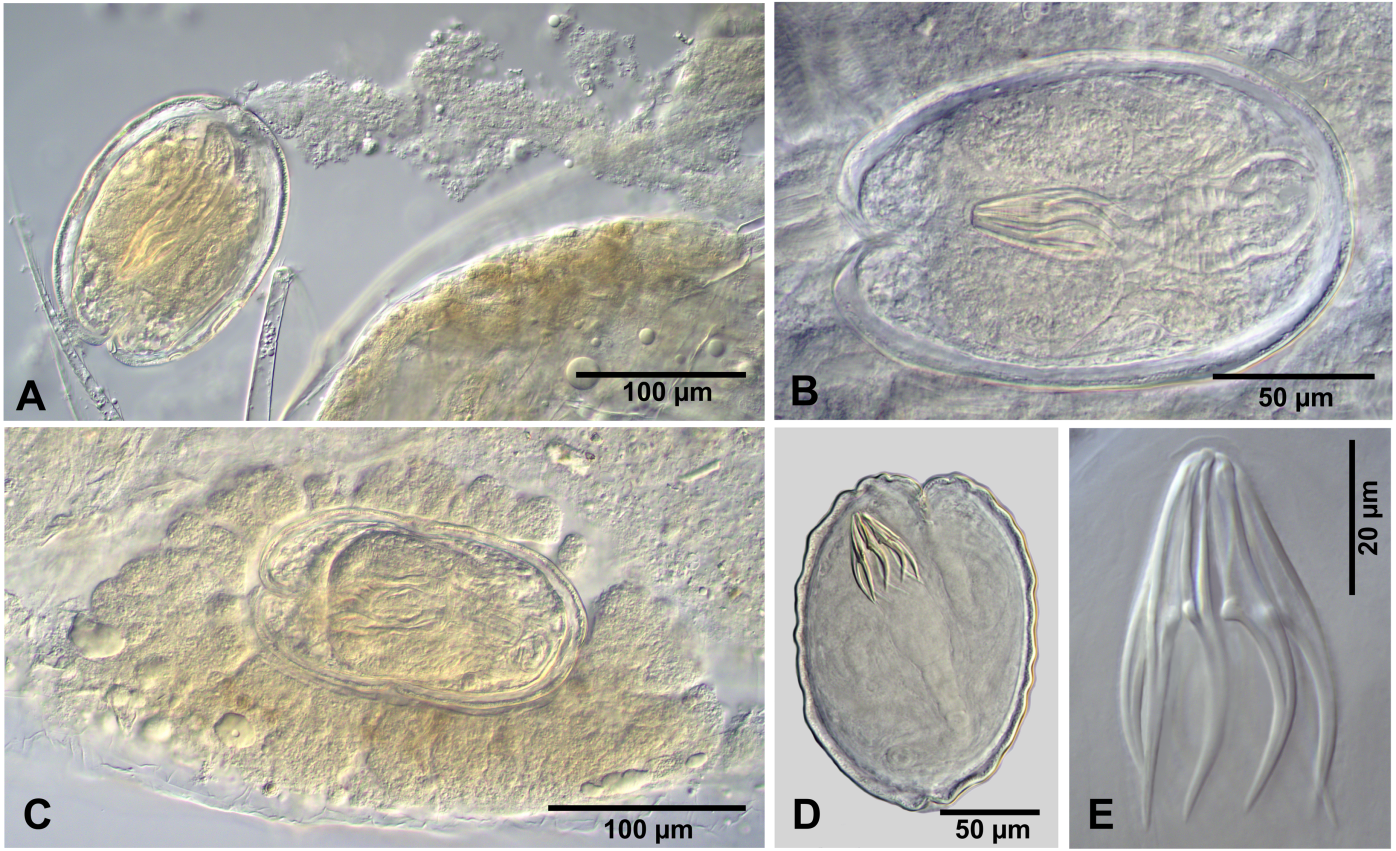
*Flamingolepis* sp. from *Artemia persimilis*, Los Cisnes lagoon. (A, C) Fully developed cysticercoid with different stage of aggregation of the cercomer (temporary glycerol mounts). (B) Fully developed cysticercoid with focus on the morphology of the scolex, rostellum and the rostellar hooks (temporary glycerol mount). (D) Isolated cysticercoid (mount in Berlese’s medium). (E) Rostellar hooks of an isolated cysticercoid (mount in Berlese’s medium).

The present finding is the first geographical record of the genus *Fimbriarioides* in Chile and in South America. The list of possible definitive hosts of *Fimbriarioides* (?) sp. in Los Cisnes and Amarga lagoons includes the representatives of Anseriformes, i.e., shelducks (Tadorninae) *Chloephaga rubidiceps* Sclater and *C. picta* (Gmelin); dabbling ducks (Anatinae) *Speculanas specularis* (King), *Anas georgica* Gmelin, *Lophonetta specularioides* (King), *Tachyeres patachonicus* (King) and *Mareca sibilatrix* Poeppig (Garay, Johnson & Franklin, 1991; Matus & Barría, 1999).

**Table 2:** Metrical data of cysticercoids of *Flamingolepis* spp. recorded in *Artemia* spp. Intermediate host and locality are also indicated. All measurements are in m except where otherwise stated.

	**Source**	Robert & Gabrion (1991)	Georgiev et al. (2005)						Maksimova (1973)		Present study		
	**Intermediate hosts**	*A. salina*	*A. parthenogenetica*						*A. salina*		*A. persimilis*		
	**Locality**	France	Spain						Kazakhstan		Chile		
	***Flamingolepis* spp.**	*F. caroli*	*F. liguloides*			*F. flamingo*			*F. dolguschini*	*F. tengizi*	*Flamingolepis* sp.		
		Range	Range	Mean	*n*	Range	Mean	*n*	Range	Range	Range	Mean	*n*
Cyst	length	300	560–810	671	14	168–270	231	14	418	180	150–163	156	8
	width	200	372–597	479	14	126–207	177	14	287	130	110–145	121	8
Scolex	length	–	358–771	614	14	141–225	182	12	254	96	113–128	122	7
	width	–	339–490	423	14	108–183	145	12	164	84	90–100	94	7
Suckers	diameter	–	181–288	233	18	45–75	66	16	121	40–42	45–53	49	14
Rostellum	length	–	446–485	465	14	108–180	119	5	–	–	100–125	114	8
	width	–	116–149	125	14	33–48	43	5	–	–	23–28	25	8
Rostellar	total length (TL)	110–130	186–201	189	15	55–61	57	15	184	53–54	45–50	49	15
hooks	length of blade (Lb)	–	105–117	110	15	28–30	29	15	102	30	24–27	25	15
	Ratio Lb/TL	–	0.56–0.60	0.58	15	0.47–0.53	0.5	15	app. 0.55	app. 0.56	0.50–0.54	0.52	15
Cercomer	length (mm)	0.40–0.50	0.62–0.81	0.71	8	6.5–7.4	–	3	0.610	–	0.60^*^	–	1

**Notes.**

* Maximum length of the longest fragment of cercomer.

***Flamingolepis* sp. (Figs. 2C, 2F, 5)**

*Description of cysticercoids* **** (metrical data based on specimens in Berlese’s medium; for some measurements see Table 2): Cercocysticercoid. Cyst elongate oval, with very thick wall, consisting of several layers; outer layer thick, with fine radial striations (Figs. 2F, 5A); numerous calcareous corpuscles situated in anterior part of cysticercoid. Scolex with conical apical protrusion and maximum width at level of suckers. Suckers unarmed, elliptical, with well-developed musculature. Rhynchus unarmed, thin-walled. Rostellar sheath thick-walled, 125–133 ×33 − 38 (130 ×36, *n* = 3), passing far beyond posterior margins of suckers; glandular cells presented in its cavity (Fig. 2F). Rostellum retractile, highly elongated, apical part with conical protrusion, posterior end usually coiled near bottom of rostellar sheath; walls thick, consisting of strong radial musculature (Figs. 2F, 5B, 5D); cavity with glandular cells. Rostellar hooks 8, skrjabinoid; each hook with long handle and very short guard; blade as long as handle or slightly longer, with smooth curvature; length of handle 21–24 (24, *n* = 15) (Figs. 2C, 5E). Cercomer considerably thick, coiled; cysticercoid with complete cercomer not available.

*Additional measurements based on specimens in temporary glycerol mounts*: Cyst 158–170 ×95 − 123 (164 ×111, *n* = 5); thickness of wall 7–12 (10, *n* = 5); scolex 118–125 ×73 − 96 (123 ×88, *n* = 5); diameter of suckers 43–50 (47, *n* = 16); rostellum 123–125 ×25 − 28 (125 ×26, *n* = 5); rostellar sheath 118–130 ×30 − 38 (123 ×35, *n* = 5); the longest available fragment of cercomer 40–75 wide and 600 long.

#### Remarks

According to the latest taxonomic revision of the family Hymenolepididae, only two genera possessing eight skrjabinoid rostellar hooks have been considered valid, i.e., *Cladogynia* Baer, 1938 and *Sobolevicanthus* Spasskii & Spasskaya, 1954 (Czaplinski in Czaplinski & Vaucher, 1994); the taxonomic concept of these two genera in the cited source postulated numerous generic synonymies based on the adoption of a few morphological criteria only (e.g., number and shape of hooks, presence of stylet in the male copulatory organ) and ignoring numerous further morphological and life-cycle characteristics, which have been used for distinguishing a number of well-defined hymenolepidid genera. By these reasons, most of the proposed synonymies have not been widely accepted and genera as *Pararetinometra* Stock & Holmes, 1982, *Retinometra* Spasskii, 1955 and *Flamingolepis* Spasskii & Spasskaya, 1954 are considered valid (Vasileva, Georgiev & Genov, 1996; Mariaux et al., 2017).

Judging on the presence of 8 skrjabinoid hooks and the extremely long retractile muscular rostellum, the cysticercoids from *A. persimilis* in Chile most closely resemble species of the genus *Flamingolepis*. Some other hymenolepidid genera parasitising aquatic birds have also similar characters, i.e., *Cladogynia*, *Retinometra, Sobolevicanthus* and *Calixolepis* Macko & Hanzelova, 1997. However, there are differences in the shape of hooks of the species of this group, despite the fact they all have been described as belonging to the “skrjabinoid type”. Compared to the hooks of the cysticercoids from Chile, the type species of *Cladogynia, C. phoeniconaiadis* (Hudson, 1934), has much bigger hooks with very long and thick base and much shorter blade (Baer, 1938). The majority of the species of the remaining three genera have hooks with a different proportion of their parts, i.e., the blades are much longer than the handles (Spasskaya, 1966; Maksimova, 1989). Moreover, the data about the life cycles of species of *Retinometra* and *Sobolevicanthus* indicated freshwater crustaceans (Copepoda, Ostracoda and Amphipoda) as intermediate hosts (Jarecka, 1960; Maksimova, 1989). *Flamingolepis* is the only hymenolepidid genus with 8 skrjabinoid hooks, which has been proven to use branchiopods as intermediate hosts. The definitive hosts are various species of Phoenicopteriformes. On this basis, we consider the above-described cysticercoids from *A. persimilis* as belonging to *Flamingolepis*.

Cysticercoids of five species of *Flamingolepis* have been recorded so far from *Artemia* spp. (Table 2). Judging on the length of hooks (45–50 µm), the cysticercoids from the present material are most similar to those of *F. flamingo* (Skrjabin, 1914) and *F. tengizi* Gvozdev & Maksimova, 1968, both parasitic in flamingos in Eurasia (Maksimova, 1989). *F. flamingo* has been recorded from *A. salina* and *A. parthenogenetica* in France (Robert & Gabrion, 1991; Sánchez et al., 2012) and Spain (Georgiev et al., 2005; Georgiev et al., 2007; Redón et al., 2011; Redón et al., 2015b; Sánchez et al., 2013). The cysticercoids from *A. persimilis* differ from those of *F. flamingo* by their greater size as well as by their bigger scolex (Table 2); furthermore, the cercomer of these cysticercoids is 40–75 µm wide while that of *F. flamingo* is only 8–12 µm wide (Georgiev et al., 2005). The cysticercoids of *F. tengizi* were described from two intermediate host species in Kazakhstan, i.e., *A. salina* (Maksimova, 1973) (Table 2) and the ostracod *Eucypris inflata* (Sars) (Gvozdev & Maksimova, 1978). The cysticercoid of *F. tengizi* described from *A. salina* differs from *Flamingolepis* sp. from Chile by the bigger cyst with thicker envelopes, the slightly bigger hooks with blades comprising more than 55% of the total hook length. The cysticercoids of the remaining three species of *Flamingolepis* differ significantly from the present material by the length of their hooks (Table 2).

In addition to the above mentioned species, which use brine shrimps in their life cycles, *Flamingolepis* includes two further species. *F. megalorchis* (Lühe, 1898) uses ostracods as intermediate hosts and has been characterised with bigger hooks compared with the present material (68–85 µm long, see Gvozdev & Maksimova, 1978). Another species is *F. chileno* Babero, Cattan & Jensen, 1981, described from *Phoenicoparrus andinus* (Philippi) in Chile (Babero, Cattan & Jensen, 1981), the only previously recorded species of *Flamingolepis* from South America. It is characterised by much bigger hooks (163–189 µm) with shape differing from that of the hooks of the cysticercoids from *A. persimilis*.

On the basis of the above mentioned remarks, we consider that the cysticercoids from *A. persimilis* from Patagonia do not fit the morphology and metrical characters of the described *Flamingolepis* spp. Most probably, these larvae belong to an undescribed species parasitizing flamingos. Its description requires examination of adult cestodes from their most probable definitive host, Chilean Flamingo *Phoenicopterus chilensis* Molina, which is abundant in hypersaline shallow lakes from Central Argentina to southern Chilean Patagonia (Battauz et al., 2013; Gibbons, Vilina & Cárcamo, 2007). The remaining two species of flamingos, Andean flamingo *Phoenicoparrus andinus* and James’ Flamingo *Phoenicoparrus jamesi* (Sclater), live in the high altitudes of the Andean plateaus of Peru, Chile, Bolivia and Argentina and the probability to be hosts of the species recorded in brine shrimps in Patagonia is low. Furthermore, *P. andinus* is primarily herbivorous, feeding on microalgae (Tobar et al., 2012).

**Figure 6:**
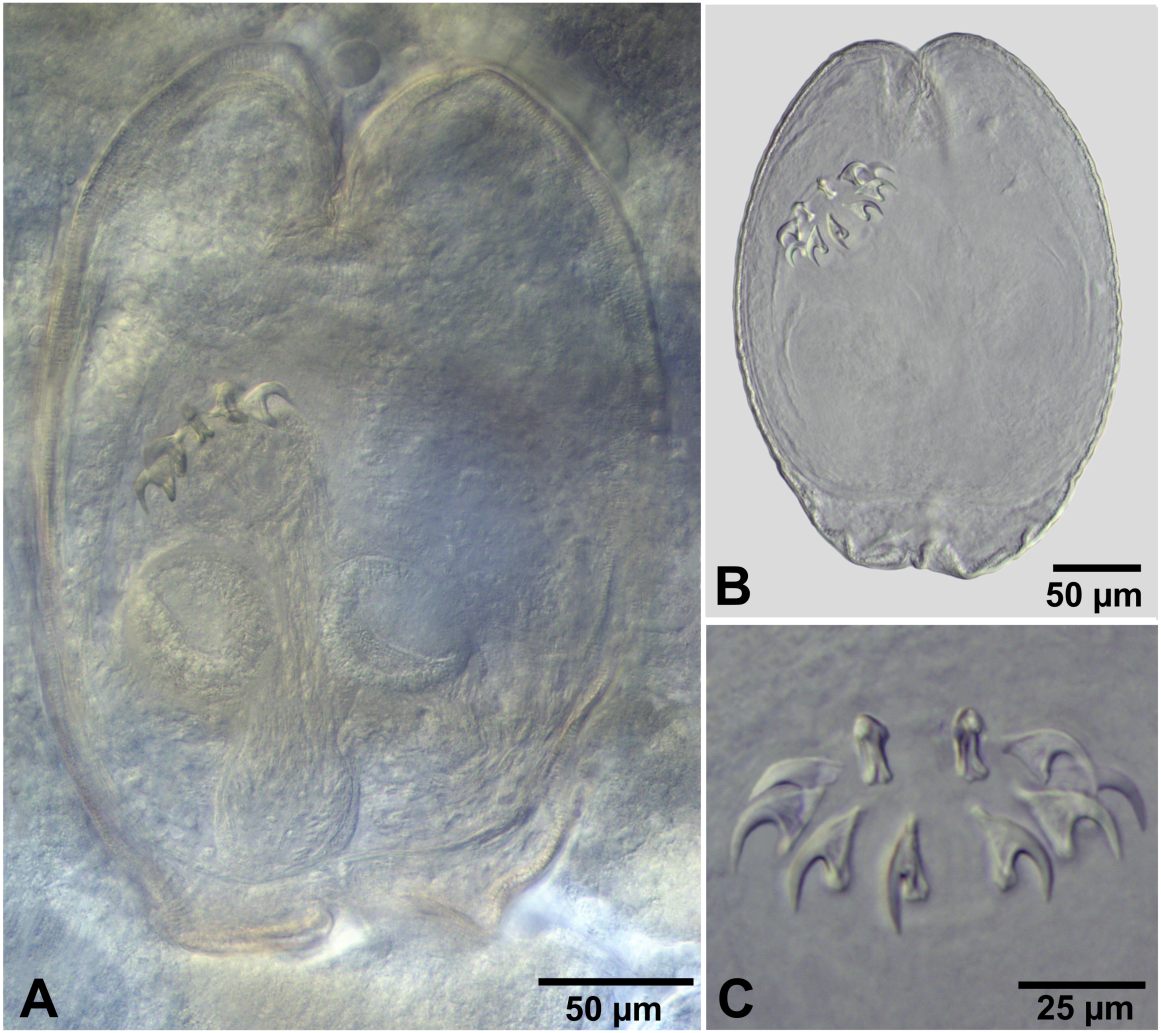
*Wardium* sp. from *Artemia persimilis*, Los Cisnes lagoon. (A) Fully developed cysticercoid (temporary glycerol mount). (B) Isolated cysticercoid (mount in Berlese’s medium). (C) Rostellar hooks of an isolated cysticercoid (mount in Berlese’s medium).

***Wardium* sp. (Figs. 2D, 6)**

*Description of cysticercoids* (metrical data based on specimens mounted in Berlese’s medium): Cercocysticercoid. Cyst lemon-shaped, thick-walled, 300–315 ×215 − 225 (*n* = 2). Scolex oval, 150–195 ×118 (*n* = 2), with maximum width at level of suckers (Fig. 6A). Suckers oval, unarmed, muscular, with diameter 43–65 (51, *n* = 6). Rhynchus short. Rostellar sheath deep, thin-walled, passing beyond level of posterior margins of suckers; 125 ×45 (*n* = 2). Rostellum invaginable, thick-walled, with apical enlargement and conically-tapering posterior part, 55–75 ×45 − 50 (*n* = 2) (Fig. 6A). Rostellar hooks aploparaksoid-like, 10 in number (Figs. 2D, 6C); blade sickle-shaped, slightly longer than guard; handle distinct, but very short, guard thick; base of hook comprised by well-developed epiphyseal thickening; total length of hooks 25–26 (25, *n* = 4), length of blade 12–13 (12, *n* = 4), length of base 18–19 (18, *n* = 4), distance between handle-tip and guard-tip 9–10 (10, *n* = 4). Calcareous corpuscles mostly concentrated in anterior part of the cyst. Cercomer 125 thick, length of longest fragment 1.4 mm. Cysticercoid with complete cercomer not available.

*Additional measurements based on specimens mounted in temporary glycerol mounts*: Cyst 300–312 ×202 − 205 (*n* = 2); scolex 150 ×120 (*n* = 1); suckers 45–64 (51, *n* = 6); rostellar sheath 113 ×48 (*n* = 1); rostellum 50 ×43 (*n* = 1).

#### Remarks

The present material corresponds to the cysticercoids of the genus *Wardium* described previously from *A. franciscana* in Great Salt Lake, USA (Redón et al., 2015a). The shape and the measurements of the cyst, the suckers and rostellum are very similar. The rostellar hooks possess the same shape and epiphyseal thickening. The length of the hooks is also similar, i.e., 25–26 µm in cysticercoids from *A. persimilis versus* 24–26 µm in *Wardium* sp. from *A. franciscana*. Based on these similarities, we identify the cysticercoids from the present material as *Wardium* sp. and consider it as conspecific to the specimens from *A. franciscana* in USA. The exact identification of the species is difficult because of the small number of cysticercoids, e.g., two larvae per each of the localities (USA and Chile). The small sample makes difficult to obtain a representative range of the measurements and the intraspecific variability. Nevertheless, the detailed comparison of the rostellar hooks of the cysticercoids of *Wardium* sp. with the known species of *Wardium* Mayhew, 1925 and *Branchiopodataenia* Bondarenko & Kontrimavichus, 2004 revealed that none of the species of these two genera can be matched with the hooks of the cysticercoids of *Wardium* sp. (see Redón et al., 2015a).

Several species of *Wardium* and *Branchiopodataenia* have been recorded so far in South America. However, none of them has rostellar hooks with shape and length similar to these of the cysticercoids from Chile. These species are: *Branchiopodataenia arctowskii* (Jarecka & Ostas, 1984) from *Larus dominicanus* Lichtenstein from the King George Island (Antarctica) (the hooks are 14–20 µm long with elongate handle, but without epiphyseal thickening, see Bondarenko & Kontrimavichus, 2004a; Bondarenko & Kontrimavichus, 2004b); *Wardium neotropicale* Deblock & Vaucher, 1997 from *Himantopus melanurus* (Vieillot) in Paraguay (hooks are 9 µm long) (see (Bondarenko & Kontrimavichus, 2006)); *W. paucispinosum* Labriola & Suriano, 2000 described from *Larus maculipennis* in Argentina (hooks are 13–18 µm long, see Labriola & Suriano, 2000); and *W. tinamoui* (Olsen, 1970) from *Nothoprocta perdicaria* (Kittlitz) in Chile (16.6 µm long, see Bondarenko & Kontrimavichus, 2006). *W. semiductilis* Szidat, 1964, recorded from *L. maculipennis* and *L. dominicanus* from Argentina (Szidat, 1964), has hooks of completely different shape (assuming diorchoid, see Szidat, 1964) and a different shape of scolex, possessing a very long rhynchus.

The definitive hosts of the species of *Wardium* and *Branchiopodataenia* include mostly aquatic birds of the order Charadriiformes (Spasskaya, 1966; Bondarenko & Kontrimavichus, 2006). The list of potential hosts at Southern Chile includes species of this order such as *Pluvianellus socialis* Gray*, Charadrius falklandicus* Latham*, C. modestus* Lichtenstein*, Vanellus chilensis* (Molina)*, Haematopus leucopodus, Larus dominicanus* (Garay, Johnson & Franklin, 1991; Matus & Barría, 1999). In addition, some of charadriiform birds migrate through both North and South America, e.g., *Limosa haemastica* (L.) (see Lepage, 2019), and they also might be hosts of these cestodes.

### Spatial and temporal variations of cestode infections

Among examined 800 brine shrimp individuals (640 adults and 160 juveniles), 92 were infected by cestodes (*P* = 11.5%). Despite most of the infected *Artemia* individuals were adults (*n* = 88), infection was also detected in 2.5% of juveniles (*n* = 4). A total of 98 cestode larvae were identified in *A. persimilis* from the two localities in the Southern Chile, with the majority of them (>87%) found at Los Cisnes lagoon (Table 3). The cysticercoids were identified as belonging to four species of four genera of the family Hymenolepididae. Two of these species, *Fimbriarioides* (?) sp. and *C. podicipina*, were recorded at both localities. The other two, *Flamingolepis* sp. and *Wardium* sp. were found at Los Cisnes lagoon only. The total intensity ranged from 1 to 3 cysticercoids; 94.6% of the infected individuals harboured one cysticercoid. The brine shrimp individuals infected with 2 and 3 cysticercoids were 4.3% (4 individuals) and 1.1% (1 individual), respectively. Single species infections were recorded in 87 host individuals. Multiple species infections were found in 5 brine shrimps: 4 individuals from Los Cisnes from the November sample (3 individuals harbouring *Flamingolepis* sp. plus *C. podicipina* and 1 individual infected with *Flamingolepis* sp. and *Fimbriarioides* (?) sp.) as well as 1 individual from Amarga from the November sample containing *Fimbriarioides* (?) sp. plus *C. podicipina*.

**Table 3:** Descriptive parameters of cestodes in brine shrimps *Artemia persimilis* from two hypersaline ecosystems in Southern Chile.

Cestode species	Number of cysticercoids	Prevalence (%)	Intensity		Abundance	
			Range	Mean ± SE	Relative (%)	Mean ± SE
**Los Cisnes lagoon**						
*Confluaria podicipina*	10	2.5	1	1.00 ± 0.00	11.6	0.025 ± 0.01
*Fimbriarioides* (?) sp.	19	4.8	1	1.00 ± 0.00	22.1	0.048 ± 0.01
*Flamingolepis* sp.	56	13.8	1–2	1.02 ± 0.01	65.1	0.140 ± 0.02
*Wardium* sp.	1	0.3	1	1.00	1.2	0.003 ± 0.00
**Overall infection**	86	20.3	1–3	1.06 ± 0.01	–	0.215 ± 0.02
**Amarga lagoon**						
*Confluaria podicipina*	2	0.5	1	1.00 ± 0.00	16.7	0.005 ± 0.00
*Fimbriarioides* (?) sp.	10	2.5	1	1.00 ± 0.00	83.3	0.025 ± 0.01
**Overall infection**	12	2.8	1–2	1.09 ± 0.02	–	0.030 ± 0.01

**Notes.**

SE standard error

The cestode community in Los Cisnes lagoon was dominated by the parasite of flamingos, *Flamingolepis* sp. (RA = 65.1%), followed by *Fimbriarioides* (?) sp. (Table 3). The later species was the most abundant in the cestode community in brine shrimps at Amarga lagoon (RA = 83.3%). *C. podicipina* was recorded exclusively in spring. *Fimbriarioides* (?) sp. was the only cestode species recorded in both seasons (Table 4).

**Table 4:** Temporal variations of cestode infection in *Artemia persimilis* in two localities from Southern Chile.

		**Los Cisnes lagoon**		**Amarga lagoon**	
**Cestode species**		Spring 2017 (*S* = 55 g/L)	Autumn 2018 (*S* = 51 g/L)	Spring 2017 (*S* = 86 g/L)	Autumn 2018 (*S* = 81 g/L)
*Confluaria podicipina*	P (%)	5.0^*^^†^	0.0	1.0	0.0
	MI ± SE	1.00 ± 0.00	0.00	1.00 ± 0.00	0.00
	MA ± SE	0.05 ± 0.02^*^^†^	0.00	0.01 ± 0.01	0.00
	RA (%)	13.5		33.3	
*Fimbriarioides* (?) sp.	P%	3.5	6.0	2.0	3.0
	MI ± SE	1.00 ± 0.00	1.00 ± 0.00	1.00 ± 0.00	1.00 ± 0.00
	MA ± SE	0.04 ± 0.01	0.06 ± 0.02	0.02 ± 0.01	0.03 ± 0.01
	RA (%)	9.5	100	66.7	100
*Flamingolepis* sp.	P%	27.5^*^^†^	0.0	0.0	0.0
	MI ± SE	1.02 ± 0.02	0.00	0.00	0.00
	MA ± SE	0.28 ± 0.03^*^^†^	0.00	0.00	0.00
	RA (%)	75.7			
*Wardium* sp.	P%	0.5	0.0	0.0	0.0
	MI ± SE	1.00	0.00	0.00	0.00
	MA ± SE	0.01 ± 0.01	0.00	0.00	0.00
	RA (%)	1.4			
**Overall infection**	P%	34.5^*^^†^	6.0	2.5	3.0
	MI ± SE	1.07 ± 0.04	1.00 ± 0.00	1.20 ± 0.03	1.00 ± 0.00
	MA ± SE	0.37 ± 0.04^*^^†^	0.06 ± 0.02	0.03 ± 0.01	0.03 ± 0.01
No. of cestode specimens		74	12	6	6
No. of *Artemia* individuals examined		200	200	200	200

**Notes.**

P (%) prevalence MI mean intensity MA mean abundance RA (%) relative abundance SE standard error S salinity measured in situ

Spatiotemporal variations at *p* level 0.05 are shown according to *Z*-test (for prevalences) and Mann–Whitney *U* test (for intensities and abundances).

* Indicates significant differences between seasons.

† Indicates significant differences between sites in the same month.

GLM performed on total cestode prevalence showed a significant effect of locality (Table S1). Values were higher at Los Cisnes lagoon than at Amarga lagoon (Table 4 and Table S1). The interaction between locality and season was also significant, denoting a higher prevalence in spring in Los Cisnes lagoon but not in Amarga lagoon (Table S1 and Table 4). GLM for *Fimbriarioides* (?) sp. showed no significant effects of either locality or season (Table S1). For *C. podicipina* (recorded exclusively in spring), a significant positive effect of Los Cisnes lagoon was detected (*B* = 1.630, χ ^2^ Wald = 4.390, *p* = 0.036).

Significant difference for prevalence of *Flamingolepis* sp. was detected between seasons in Los Cisnes lagoon, registering higher values in spring (*Z* = 7.840, *p* < 0.001; Table 4). The overall abundance and the abundance of *C. podicipina* and *Flamingolepis* sp. were also significantly higher in spring than in autumn at Los Cisnes lagoon (Mann–Whitney tests, *p* < 0.001; Table 4 and Table S2). However, no seasonal differences were detected in Amarga lagoon for either total abundance or any cestode species (Mann–Whitney tests, *p* > 0.157; Table S2). Comparing between both localities in the same season, significant differences were detected for the prevalence of *Flamingolepis* sp. (*Z* = 7.840, *p* < 0.001) as well as for the overall abundance (Mann–Whitney, *p* < 0.001; Table S2) and the abundance of *C. podicipina* and *Flamingolepis* sp. in spring (Mann–Whitney test, *p* < 0.019; Table S2), with lower values in Amarga lagoon (Table 4). In contrast, for *Fimbriarioides* (?) sp., differences in abundance between localities were not statistically significant (Mann–Whitney test, *p* > 0.148, Table S2 and Table 4).

No seasonal variations were detected for the intensity of the overall infection in Los Cisnes lagoon (Mann–Whitney test, *U* = 390, *p* = 0.395) or Amarga lagoon (Mann–Whitney test, *U* = 12, *p* = 0.273) nor between localities in the same season (Mann–Whitney tests, *p* > 0.302; Table 4).

## Discussion

### Species composition of cestode larvae in relation to the avian communities

The present study provides the first evidence that *A. persimilis* participates in the life cycles of four cestode species of the family Hymenolepididae. These include: *C. podicipina*, a specific parasite of grebes; *Flamingolepis* sp., an unidentified cestode parasitic in flamingos; *Fimbriarioides* (?) sp. and *Wardium* sp., two unidentified species that could be parasitic in ducks or charadriiform birds.

Previous studies in the Western Mediterranean and North America have revealed that the prevalence of avian cestodes in brine shrimps may depend on the seasonal presence of definitive hosts at certain hypersaline wetlands (Georgiev et al., 2005; Georgiev et al., 2014; Sánchez et al., 2013; Redón et al., 2015a). On this basis, we could expect that the peculiarities of parasite communities in brine shrimps from Los Cisnes and Amarga lagoons are due to the differences between the avian communities of the two wetlands. Unfortunately, the information on the population dynamics of aquatic birds at both sampling sites in the Southern Chile is very limited.

Los Cisnes lagoon is an area with diverse habitats (Fuentes-González & Gajardo, 2017). This wetland is formed by a lagoon with a long peninsula protruded into it and surrounded by several temporary shallow water bodies and muddy areas used as feeding places for charadriiforms such as White-rumped Sandpiper *Calidris fuscicollis*, Baird’s Sandpiper *C. bairdii*, Two-banded Plover *Charadrius falklandicus*, Rufous-chested Plover *C. modestus* and the Magellanic Plover *Pluvianellus socialis*. Other frequent birds in the lagoon are *Podiceps occipitalis, P. gallardoi* and *Phoenicopterus chilensis*, particularly** in winter and spring (CONAF, 2014; Roesler, 2015). The Amarga lagoon is a shallow hypersaline lake at the eastern border of the Torres del Paine National Park. The following species are among the most abundant birds year-round: Black-necked Swan *Cygnus melancoryphus*, Coscoroba Swan *Coscoroba coscoroba*, Upland Goose *Chloephaga picta*, several species of dabbling ducks, grebes *Rollandia roland* and *Podiceps occipitalis* (see Garay, Johnson & Franklin, 1991) as well as the flamingo *Phoenicopterus chilensis*, especially in spring** (Campos et al., 1996; Matus & Barría, 1999). This avian diversity at the two examined wetlands is in agreement with the species composition of cestode larvae in *A. persimilis* found in the course of the present study. The most abundant cestode species in brine shrimps in Los Cisnes lagoon are *Flamingolepis* sp. (parasite of flamingos) and *Fimbriarioides* (?) sp., probably parasitic in shelducks (Tadorninae) or dabbling ducks (Anatinae). Data from dietary studies confirm that the most probable definitive host of *Flamingolepis* sp. is the Chilean Flamingo, which is feeding mainly on brine shrimps (Hurlbert, López & Keith, 1984) and other invertebrates (such as copepods, cladocers, rotifers, ostracods), and to a lesser extent on bacteria and microalgae (Polla et al., 2018).

The specific parasite of grebes *C. podicipina* was present at both sampling sites. However, comparing with data from previous studies in Spain and USA (Georgiev et al., 2005; Redón et al., 2011; Redón et al., 2015a), the prevalence, mean abundance and mean intensity of *C. podicipina* from *A. persimilis* in Chile are lower. For example, the prevalence in *A. persimilis* from Los Cisnes is 2.5% *versus* 28.7% in a population from *A. parthenogenetica* from Spain (Redón et al., 2011) and 27.4–40.6% in *A. franciscana* from the Great Salt Lake (Redón et al., 2015a). These dissimilarities could be due to differences in the abundance of the grebe populations at the sampling sites, *P. nigricollis* in the Holarctic and, most probably, *P. occipitalis* in the Neotropical Region. The high infection parameters at Great Salt Lake can be explained by the fact that the second largest staging population of *P. nigricollis* in North America (over 1 million grebes) occurs there (Redón et al., 2015a).

The low prevalence of larvae of parasites of charadriiform birds such as *Wardium* sp. as well as the lack of cysticercoids of the family Dilepididae were unexpected. However, further studies including (austral) summer and winter months might provide more parasitological data on cestodes of charadriforms since many Nearctic species arrive to South America for wintering during austral summer (i.e., from December to February). With the diversity of shores and shallow waters at both sampling sites and the variety of shorebirds inhabiting them (Garay, Johnson & Franklin, 1991; Matus & Barría, 1999), it could be expected that *A*. *persimilis* may play a role as an intermediate host for more cestode species, especially dilepidids occurring as adults in shorebirds (Georgiev et al., 2005; Redón et al., 2015a).

### Spatial and temporal variations of cestode communities and bird communities

We have found that the two studied localities significantly differ concerning the overall cestode infection in *A. persimilis*, with higher prevalence of infection in Los Cisnes lagoon. Seasonal effects on the prevalence have been detected only for Los Cisnes lagoon, there being a significant locality ×season interaction. The present study has revealed both spatial and temporal variations in the prevalence and mean abundance of two species, *Flamingolepis* sp. and *C. podicipina* (Table 4), and therefore variations of the overall cestode abundance. The seasonal abundance of *Flamingolepis* sp. in spring samples from Los Cisnes lagoon may be explained by the abundance of Chilean flamingos, which are one of the main *Artemia* predators in Patagonian hypersaline wetlands (Campos et al., 1996). It is also considered as a common summer resident at Torres del Paine National Park (Garay, Johnson & Franklin, 1991). *C. podicipina* has been recorded only in spring at both localities, a fact suggesting the presence of grebes in a certain short period only. The most probable definitive host, *P. occipitalis*, is a resident bird at the two localities but it migrates to the Atlantic Coast and to Paraguay in non-breeding seasons (Matus & Barría, 1999), which might explain the lack of cysticercoids of *C. podicipina* in the autumn samples. Cysticercoids of *Fimbriarioides* (?) sp. were recorded at both localities and both seasons; its infection parameters did not demonstrate apparent seasonal trends (Table 4). This could be explained by the presence of waterfowl during the entire annual cycle at both sampling sites, especially the shelducks *Cloephaga picta* and *C. poliocephala*, as well as various dabbling ducks (Garay, Johnson & Franklin, 1991; Matus & Barría, 1999).

The limited amount of information on the population dynamics of aquatic birds at both sites demands further studies for a more comprehensive knowledge of host-parasite interactions in the “cestode –*Artemia* –aquatic birds” system in the hypersaline habitats in the austral part of Chile.

### Contribution to the cestode fauna of the Neotropical aquatic birds

Previous surveys on helminth parasites from aquatic birds in southern South America are scarce, with a few studies from the last decades including parasites of gulls (*Larus dominicanus* and *L. maculipennis*), cormorants (*Phalacrocorax olivaceus*), grebes (*Podiceps occipitalis, P. major* and *P. gallardoi*) and swans (*Cygnus melanocoryphus*) (see Torres et al., 1991; Torres et al., 1993; González-Acuña et al., 2009; González-Acuña et al., 2010; González-Acuña et al., 2017; Núñez et al., 2017b). The available data on the helminth fauna associated to Chilean Flamingo are very limited, consisting of two reports of nematodes from captive birds in the San Francisco Zoo (Fox et al., 1974) and two further nematodes from Central Argentina (Núñez et al., 2017a). The data about cestodes from grebes in Chile are limited to a few cestode taxa recorded in several grebe species (for review, see Storer, 2000). The Neotropical Region has been considered as not sufficiently studied area in terms of the diversity of the avian cyclophyllidean cestodes (Mariaux et al., 2017). In this sense, our results contribute to the knowledge of the diversity of cestodes of aquatic birds in the region, providing the first geographical records of *C. podicipina* and the genus *Fimbriarioides* for the Neotropics*.* We also believe that *Flamingolepis* sp. from *A. persimilis* represents a new undescribed species, most probably a specific parasite of the Chilean Flamingo. In addition, the present work shows that hypersaline wetlands and their most conspicuous inhabitant (*Artemia*) are an excellent model system for parasitological studies and a useful tool to evaluate and monitor aquatic biodiversity (including hosts and parasites) or to explore the “hidden fauna” (avian endoparasites). The Magallanes and Chilean Antarctica Region is a pristine area and its hypersaline wetlands provide habitat for diverse aquatic birds. Thus we could expect that further studies on parasites of various groups of crustaceans can also contribute to increase the knowledge of avian cestode diversity in the region.

## Conclusions

The present study provides the first evidence for the transmission of avian cestode parasites using brine shrimps as intermediate hosts in the Neotropical Region. It demonstrates the role of *A. persimilis* in the life cycles of at least four cestode species of the family Hymenolepididae parasitic in aquatic birds. These are specific parasites of grebes and flamingos as well as two unidentified species, most probably parasitic in ducks or charadriiform birds. Despite the lack of data on the adult cestodes, our finding represents the first data of the occurrence of a specific cestode parasite of flamingo (*Flamingolepis* sp.) transmitted via brine shrimps in hypersaline ecosystems of the Southern Chilean Patagonia. A more comprehensive research of wildlife parasite diversity of aquatic birds in South America is needed for better knowledge of the parasite life cycles and the factors affecting the host-parasite interactions.

##  Supplemental Information

10.7717/peerj.7395/supp-1 Table S1 Results from GLM of the effects of season, locality and their interaction on the prevalence of cestode infection using a binomial error distribution and a logit link function (0 = non infected; 1 = infected) Localities are Los Cisnes and Amarga lagoons. Seasons are spring and autumn. Total cestode prevalence and prevalence for *Fimbriarioides* (?) sp. were analysed. Estimates for “Amarga lagoon”, “spring” and Los Cisnes * spring, Amarga * autumn, and Amarga * spring interactions are not included because they were aliased but they are effectively zero. Significant effects are indicated in bold.

10.7717/peerj.7395/supp-2 Table S2 Results for Mann-Whitney tests for the abundance of cestodes in *A. persimilis* between localities and seasons Abundance for the overall infection and for each cestode species is presented. CIS, Los Cisnes lagoon; AMA, Amarga lagoon; S, spring; A, autumn. Significant *p*-values at 0.05 level are indicated in bold.

10.7717/peerj.7395/supp-3 File S1 Metrical data of cestode cysticercoids found in the crustacean intermediate host *Artemia persimilis* in Chile

10.7717/peerj.7395/supp-4 File S2 Raw data of cestode infection in the crustacean intermediate host *Artemia persimilis* in Chile

## Additional Information and Declarations

### Field Study Permissions

The following information was supplied relating to field study approvals (i.e., approving body and any reference numbers): Field work was authorized by the National Forest Corporation of Chile (CONAF), Management of Protected Wild Areas, Department of Conservation of Biological Diversity (Permission number 025/2017).

### Data Availability

The following information was supplied regarding data availability: The raw measurements are available in the Supplemental Files. The raw data shows metrical data of all cestode cysticercoids used for morphological descriptions and parasite identification (File S1), and raw infection data used to quantify infection parameters and for statistical analysis (File S2).
