# Supplementary material for: First report of cestode infection in the crustacean Artemia persimilis from Southern Chilean Patagonia and its relation with the Neotropical aquatic birds
Source: PeerJ. 2019 Aug 6;7:e7395. doi: 10.7717/peerj.7395 (PMC6688596; doi:10.7717/peerj.7395)
Supplement: Table S1 — Localities are Los Cisnes and Amarga lagoons. Seasons are spring and autumn. Total cestode prevalence and prevalence for Fimbriarioides (?) sp. were analysed. Estimates for “Amarga lagoon”, “spring” and Los Cisnes * spring, Amarga * autumn, and Amarga * spring interactions are not included because they were aliased but they are effectively zero. Significant effects are indicated in bold. [file peerj-07-7395-s001.docx]

|  | **Estimate** | **SE** | **Confidence Interval Wald 95%** | | **Exp(B)** | **Wald Stat** | **df** | **Pr (>\|w\|)** |
| --- | --- | --- | --- | --- | --- | --- | --- | --- |
|  |  |  | **Lower** | **Upper** |  |  |  |  |
| *a) Total Prevalence* |  |  |  |  |  |  |  |  |
| Intercept | -3.664 | 0.453 | -4.551 | -2.776 | 0.026 | 65.431 | 1 | **0.000** |
| Locality (Los Cisnes) | 3.022 | 0.477 | 2.088 | 3.957 | 20.542 | 40.199 | 1 | **0.000** |
| Season (Autumn) | 0.187 | 0.614 | -1.016 | 1.391 | 1.206 | 0.093 | 1 | 0.760 |
| Locality*Season (Los Cisnes*Autumn) | -2.298 | 0.698 | -3.667 | -0.929 | 0.100 | 10.826 | 1 | **0.001** |
| *b) Fimbriarioides* (?) sp. |  |  |  |  |  |  |  |  |
| Intercept | -3.892 | 0.5051 | -4.882 | -2.902 | 0.020 | 59.373 | 1 | **0.000** |
| Locality (Los Cisnes) | 0.575 | 0.6349 | -0.669 | 1.819 | 1.777 | 0.820 | 1 | 0.365 |
| Season (Autumn) | 0.416 | 0.6534 | -0.865 | 1.696 | 1.515 | 0.405 | 1 | 0.525 |
| Locality*Season (Los Cisnes*Autumn) | 0.150 | 0.8146 | -1.447 | 1.746 | 1.161 | 0.034 | 1 | 0.854 |
