# Supplementary material for: First report of cestode infection in the crustacean Artemia persimilis from Southern Chilean Patagonia and its relation with the Neotropical aquatic birds
Source: PeerJ. 2019 Aug 6;7:e7395. doi: 10.7717/peerj.7395 (PMC6688596; doi:10.7717/peerj.7395)
Supplement: Table S2 — Abundance for the overall infection and for each cestode species is presented. CIS, Los Cisnes lagoon; AMA, Amarga lagoon; S, spring; A, autumn. Significant p-values at 0.05 level are indicated in bold. [file peerj-07-7395-s002.docx]

|  | **Total Abundance** | ***C. podicipina*** | ***Fimbriarioides* (?) sp.** | ***Flamingolepis* sp.** | ***Wardium* sp.** |
| --- | --- | --- | --- | --- | --- |
| **CIS S-CIS A** |  |  |  |  |  |
| Mann-Whitney *U* | 14276 | 19000 | 19500 | 14500 | 19900 |
| Z | -7.104 | -3.199 | -1.174 | -7.974 | -1.000 |
| *p*-value | **0.000** | **0.001** | 0.240 | **0.000** | 0.317 |
| **AMA S-AMA A** |  |  |  |  |  |
| Mann-Whitney *U* | 19903 | 19800 | 19800 | - | - |
| Z | -0.296 | -1.416 | -0.640 | - | - |
| *p*-value | 0.767 | 0.157 | 0.522 | - | - |
| **CIS S-AMA S** |  |  |  |  |  |
| Mann-Whitney *U* | 13624 | 19200 | 19700 | 14500 | 19900 |
| Z | -8.189 | -2.342 | -0.916 | -7.974 | -1.000 |
| *p*-value | **0.000** | **0.019** | 0.360 | **0.000** | 0.317 |
| **CIS A-AMA A** |  |  |  |  |  |
| Mann-Whitney *U* | 19400 | - | 19400 | - | - |
| Z | -1.445 | - | -1.445 | - | - |
| *p*-value | 0.148 | - | 0.148 | - | - |
